# Glucocerebrosidase modulates cognitive and motor activities in murine models of Parkinson’s disease

**DOI:** 10.1093/hmg/ddw124

**Published:** 2016-04-28

**Authors:** Edward Rockenstein, Jennifer Clarke, Catherine Viel, Nicholas Panarello, Christopher M. Treleaven, Changyoun Kim, Brian Spencer, Anthony Adame, Hyejung Park, James C. Dodge, Seng H. Cheng, Lamya S. Shihabuddin, E. Masliah, S. Pablo Sardi

**Affiliations:** 1 Neuroscience Department, University of California San Diego, La Jolla, CA 92093, USA; 2 Sanofi Genzyme, Framingham, MA 01701, USA; 3 Pathology Department, University of California San Diego, La Jolla, CA 92093, USA

## Abstract

Mutations in *GBA1*, the gene encoding glucocerebrosidase, are associated with an enhanced risk of developing synucleinopathies such as Parkinson’s disease (PD) and dementia with Lewy bodies. A higher prevalence and increased severity of motor and non-motor symptoms is observed in PD patients harboring mutant *GBA1* alleles, suggesting a link between the gene or gene product and disease development. Interestingly, PD patients without mutations in *GBA1* also exhibit lower levels of glucocerebrosidase activity in the central nervous system (CNS), implicating this lysosomal enzyme in disease pathogenesis. Here, we investigated whether modulation of glucocerebrosidase activity in murine models of synucleinopathy (expressing wild type *Gba1*) affected α-synuclein accumulation and behavioral phenotypes. Partial inhibition of glucocerebrosidase activity in *PrP-A53T-SNCA* mice using the covalent inhibitor conduritol-B-epoxide induced a profound increase in soluble α-synuclein in the CNS and exacerbated cognitive and motor deficits. Conversely, augmenting glucocerebrosidase activity in the *Thy1-SNCA* mouse model of PD delayed the progression of synucleinopathy. Adeno-associated virus-mediated expression of glucocerebrosidase in the *Thy1-SNCA* mouse striatum led to decrease in the levels of the proteinase K-resistant fraction of α-synuclein, amelioration of behavioral aberrations and protection from loss of striatal dopaminergic markers. These data indicate that increasing glucocerebrosidase activity can influence α-synuclein homeostasis, thereby reducing the progression of synucleinopathies. This study provides robust *in vivo* evidence that augmentation of CNS glucocerebrosidase activity is a potential therapeutic strategy for PD, regardless of the mutation status of *GBA1*.

## Introduction

Parkinson’s disease (PD) and dementia with Lewy bodies (DLB) are neurodegenerative diseases characterized by clinical features of Parkinsonism and cognitive impairment. They are typically associated with the pathological development of neuronal α-synuclein-positive Lewy bodies and Lewy neurites (1). Recently, a strong association was demonstrated between mutations in the *GBA1* gene (OMIM 606463), which encodes the lysosomal enzyme glucocerebrosidase, and the development of these synucleinopathies. Pathogenetic mutations in both alleles of *GBA1* cause the lysosomal storage disorder Gaucher disease. Carriers of a single mutant *GBA1* allele are unaffected by Gaucher disease. However, heterozygous mutations in *GBA1* have been associated with a significantly heightened risk of developing synucleinopathies (2–5). While the general clinical and pathological phenotypes of PD patients with *GBA1* mutations are largely indistinguishable from those of individuals with sporadic PD, genetic variation in *GBA1* has emerged as a significant feature impacting the natural history of PD (6). Patients who harbor *GBA1* mutations present with a higher prevalence and severity of motor and non-motor symptoms (6–15).

The role of *GBA1* mutations in the pathogenesis of synucleinopathies is not fully understood, but experimental data suggest that there may be a direct relationship between the level of glucocerebrosidase activity and α-synuclein proteostasis (16). Both loss-of-function and toxic gain-of-function mutations in *GBA1* have been proposed as possible contributing factors (16). The significance of glucocerebrosidase activity is underscored by the fact that *GBA1* null mutations in humans (e.g. 84GG) are associated with a higher risk of developing PD (2,17). In addition, carriers of severe *GBA1* mutations (i.e. IVS 2 + 1, 84GG, D409H) exhibit a substantially decreased age of onset of PD compared with carriers with milder mutations associated higher residual enzyme activity (i.e. N370S) (17). Patients with homozygous or compound heterozygous mutations in *GBA1* have the earliest age of onset (17). The gene dosage effect of *GBA1* on the onset and age-specific risk of developing PD has now been confirmed by several independent laboratories (18–23).

Additional evidence for a role of glucocerebrosidase in α-synuclein homeostasis has also been generated in animal studies. Several independent groups have reported increased accumulation of α-synuclein in the brains of mouse models of Gaucher disease with different pathogenetic *Gba1* mutations (24–29). Importantly, studies have shown that increasing glucocerebrosidase levels in the central nervous system (CNS) of these animals reduce the extent of α-synuclein-mediated pathology. Expression of WT glucocerebrosidase reduced the accumulation of α-synuclein in the hippocampi of mouse models of Gaucher disease and synucleinopathy (26,30).

Hence, although the precise mechanistic basis of *GBA1*-mediated PD remains unknown, evidence suggests that glucocerebrosidase haploinsufficiency, as a result of *GBA1* mutations, can interfere with α-synuclein processing and contribute to the pathological accumulation of the protein (16). This study supports this premise by demonstrating that a partial reduction in CNS glucocerebrosidase activity in *PrP–A53T–SNCA* mice led to accumulation of undergraded lipids, misprocessing of α-synuclein and neuronal transcriptional dysregulation. Importantly, these aberrations were associated with exacerbation of motor and cognitive phenotypes. In contrast, augmenting glucocerebrosidase activity within the striatum of the *Thy1-SNCA* mice reduced α-synuclein pathology and, most notably, ameliorated behavioral deficits and protected against the loss of dopaminergic terminals. Together, the results demonstrated that modulation of glucocerebrosidase activity in the CNS of two murine models of PD, independent of the mutation status of *Gba1*, could affect α-synuclein homeostasis and, in turn, PD progression.

## Results

### Pharmacological inhibition of glucocerebrosidase activity promotes the accumulation of lipids and α-synuclein in the brain

**Figure 1.**
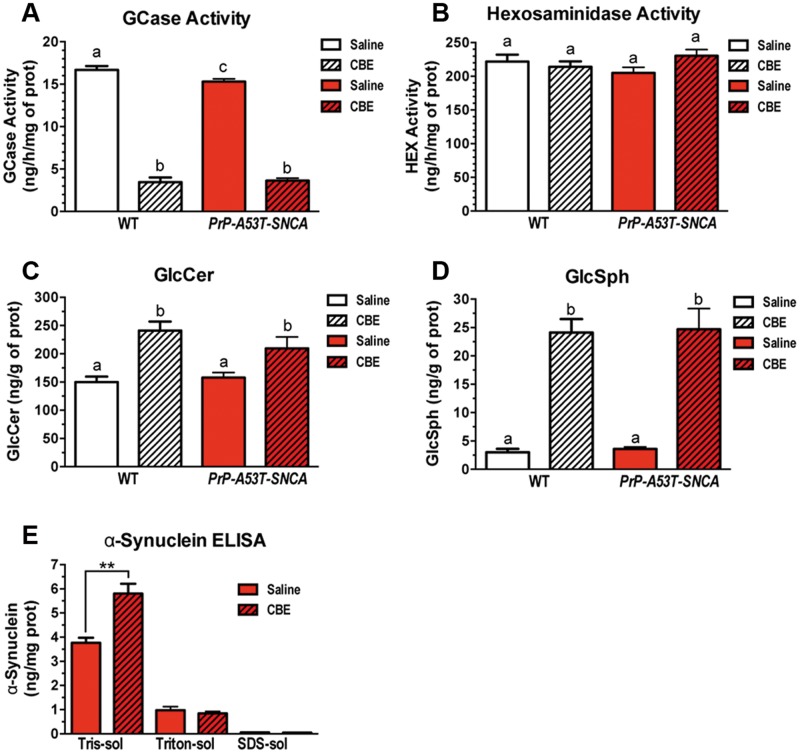
CBE-mediated partial inhibition of glucocerebrosidase activity promotes increased accumulation of glucosylceramide, glucosylsphingosine and α-synuclein in the CNS. Two-month-old WT (white bars, *n* = 10 per group) and *PrP-A53T-SNCA* (red bars, *n* = 12 per group) mice were injected with saline (solid columns) or the glucocerebrosidase inhibitor CBE (100 mg/kg, *ip*, three times per week, hatched columns). (**A**) Brains from *PrP-A53T-SNCA* mice showed a small but significant reduction in glucocerebrosidase activity when compared with WT mice, as previously described (26). This activity was significantly reduced by CBE treatment in both WT and *PrP-A53T-SNCA* animals, confirming the ability of the inhibitor to achieve widespread brain distribution. (**B**) Treatment with the glucocerebrosidase inhibitor did not affect the activity of hexosaminidase, another lysosomal hydrolase. (**C, D**) CBE-mediated inhibition of glucocerebrosidase promoted accumulation of glucosylceramide and glucosylsphingosine in the CNS of both WT and *PrP-A53T-SNCA* mice (white and red hatched bars, *p *< 0.01). (**E**) Inhibition of glucocerebrosidase activity also promoted the accumulation of α-synuclein in the cytosolic fraction (Tris-soluble, non-membrane-associated; *p *< 0.01). All data are presented as the mean ± SEM. Bars with different letters are significantly different from each other (*p *< 0.05).

*PrP-A53T-SNCA* transgenic mice express mutant human A53T α-synuclein under the murine PrP promoter and harbor wild-type *Gba1* alleles (31). Cortical lysates of *PrP-A53T-SNCA* mice have decreased glucocerebrosidase activity (30), similar to that observed in the brains of sporadic PD patients expressing WT *GBA1* alleles (32,33). Here, we replicated these findings in the brains of *PrP-A53T-SNCA* mice, as illustrated by the modest but significant reduction in glucocerebrosidase activity compared to WT controls (Fig. 1A; WT saline: 16.7 ± 0.5 ng/h/mg of protein, *PrP-A53T-SNCA* saline: 15.3 ± 0.3 ng/h/mg of protein; *p *< 0.05).

To determine if a chemically induced reduction in glucocerebrosidase activity in the CNS of mice that do not harbor mutations in *Gba1* impacted their pathological and behavioral features, young adult WT and *PrP-A53T-SNCA* mice were treated with conduritol-B-epoxide (CBE, 100 mg/kg, *ip*, 10 weeks). This irreversible inhibitor was administered three times per week to promote a partial blockade of the glucocerebrosidase activity as noted in patients carrying *GBA1* heterozygous mutations (32). Pharmacological inhibition with CBE decreased the brain glucocerebrosidase activity to ∼20% of that of the WT controls (Fig. 1A; WT CBE: 3.5 ± 0.5 ng/h/mg of protein, *p *< 0.05 versus WT saline; *PrP-A53T-SNCA* CBE: 3.6 ± 0.3 ng/h/mg of protein, *p *< 0.05 versus *PrP-A53T-SNCA* saline). On the other hand, CBE-treatment did not alter the activity of the lysosomal hydrolase hexosaminidase, demonstrating the specificity of the glucocerebrosidase pharmacological inhibitor (Fig. 1B). The brain levels of the glucocerebrosidase’s substrates, glucosylceramide and glucosylsphingosine, were dramatically increased, confirming the efficacy of the pharmacological inhibitor in the CNS (Fig. 1C and D; GlcCer: WT CBE 1.6-fold increase versus WT saline; *PrP-A53T-SNCA* CBE: 1.3-fold increase versus *PrP-A53T-SNCA* saline; GlcSph: WT CBE 7.8-fold increase versus WT saline; *PrP-A53T-SNCA* CBE: 6.5-fold increase versus *PrP-A53T-SNCA* saline).

We next evaluated whether decreasing the glucocerebrosidase activity promoted the accumulation of mutant human α-synuclein in the brains of transgenic mice. Cortical tissue homogenates of *PrP-A53T-SNCA* mice were subjected to serial fractionation to separate the cytosolic-soluble, membrane-associated, and insoluble forms of α-synuclein (30). The levels of cytosolic soluble α-synuclein were significantly increased by CBE treatment as quantified by ELISA (Fig. 1E; *PrP-A53T-SNCA* saline: 3.8 ± 0.2 ng/mg of protein, *PrP-A53T-SNCA* CBE: 5.8 ± 0.4 ng/mg of protein, *p *< 0.01). In contrast, the levels of membrane-associated α-synuclein and the insoluble fraction remained unchanged by 10 weeks of CBE treatment (Fig. 1E). Importantly, the rise in α-synuclein protein level was not because of increased *SNCA* transcription, as its mRNA levels remained unchanged (*SNCA* mRNA: *PrP-A53T-SNCA* CBE 1.07 ± 0.05 versus *PrP-A53T-SNCA* saline, *p *= 0.42). These data indicated that a reduction in glucocerebrosidase activity (independent of mutations in *GBA1*) could modulate α-synuclein processing and suggested that normal levels of glucocerebrosidase activity are required to maintain a steady-state level of α-synuclein *in vivo*. This finding is consistent with previous data demonstrating that increasing glucocerebrosidase activity in *PrP-A53T-SNCA* mice reduced soluble cytosolic α-synuclein levels but did not affect the membrane-associated α-synuclein or the insoluble fractions (30).

### Pharmacological inhibition of glucocerebrosidase activity negatively affects motor skills and cognitive function

To determine if a reduction in glucocerebrosidase activity (that is independent of mutations in *Gba1*) in the CNS of mice impacted their behavioral features, young adult WT and *PrP-A53T-SNCA* mice were treated with CBE (100 mg/kg, *ip*, 10 weeks) or saline. Motor ability was determined by examining the fine motor skills (i.e. pole test and nest-building test) and gross motor function (i.e. rotarod and ambulatory competence) of the mice after 8 and 9 weeks of CBE treatment, respectively. Gross visual inspection failed to detect any overt motor dysfunction such as ataxia, tremor or paralysis during the study period.

**Figure 2.**
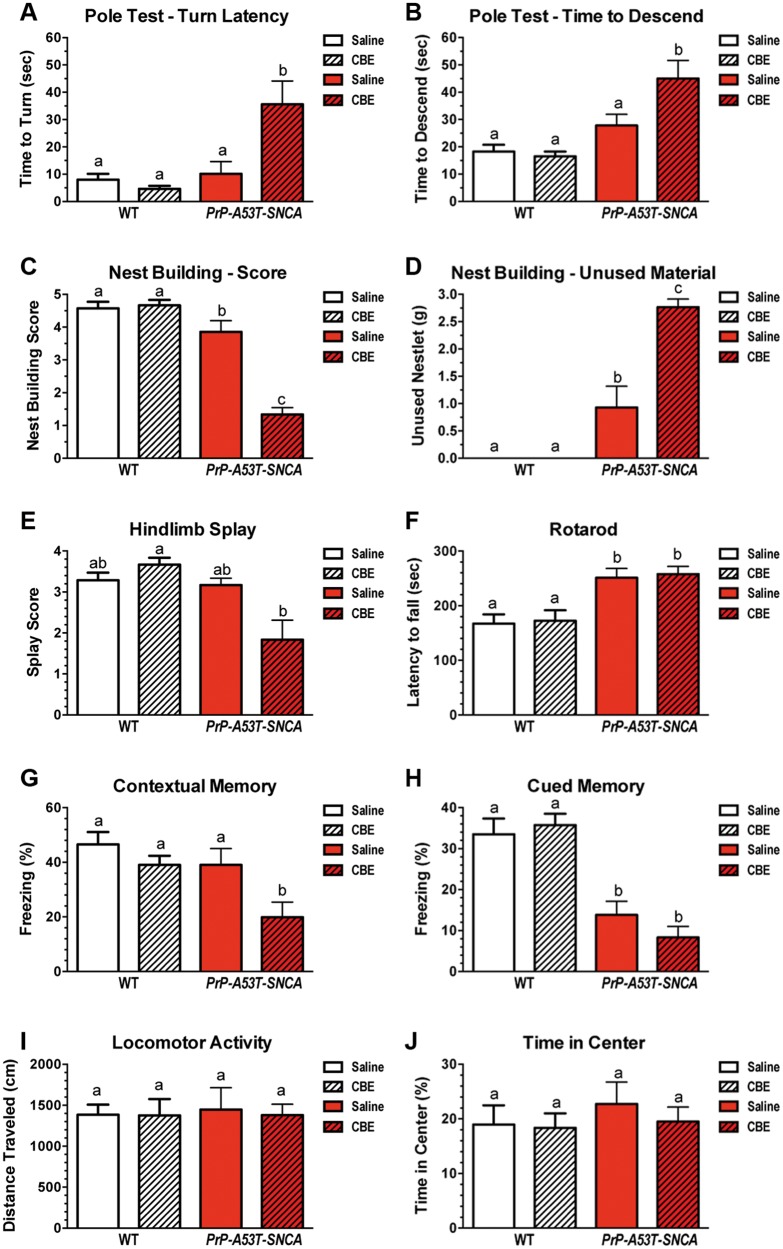
Reducing glucocerebrosidase activity in the CNS of A53T-α-synuclein mice exacerbates motor and cognitive deficiencies. Two-month-old WT (white bars, *n* = 10 per group) and *PrP-A53T-SNCA* (red bars, *n* = 12 per group) mice were injected three times per week with saline (solid bars) or 100 mg/kg CBE (hatched bars) for 10 weeks. Fine motor skills were assessed using the pole (**A, B**), nest-building (**C, D**), and hindlimb splay (**E**) tests. Treatment of WT mice with CBE had no effect on the results of any of these behavioral assessments when compared with the saline-treated controls. Saline-treated *PrP-A53T-SNCA* mice showed a mild deficit in nest-building scores compared to WT mice (C). This finding is corroborated by the finding that the control *PrP-A53T-SNCA* animals had a greater amount of unused nestlet material (D, solid red bar). Glucocerebrosidase inhibition caused a marked deterioration in fine motor skills after 8 weeks of treatment with CBE. CBE-treated *PrP-A53T-SNCA* mice displayed significantly longer turn and descent latencies than any of the other cohorts (A-B, red hatched bar, *p *< 0.05). CBE treatment also worsened the nest-building scores of *PrP-A53T-SNCA* mice (C, red hatched bar, *p *< 0.05). This result was corroborated by the finding of large amounts of unused nestlet material with this group of animals (D, red hatched bar, *p *< 0.05). CBE treatment caused a hind-limb splay deficit in *PrP-A53T-SNCA* mice (E, red hatched bar, *p *< 0.05). *PrP-A53T-SNCA* mice displayed improved rotarod latencies compared with WT controls (**F**, white versus red bars, *p *< 0.01). The ability of either WT or *PrP-A53T-SNCA* mice to remain on the rotating rod was unaffected by CBE-mediated inhibition of glucocerebrosidase (F, hatched bars, *p *> 0.05). The cognitive status of WT and *PrP-A53T-SNCA* mice was evaluated by monitoring their conditioned fear responses. *PrP-A53T-SNCA* mice showed no contextual memory deficits at 4 months of age when compared with age-matched WT mice. However, their hippocampal memory was affected upon treatment with CBE (**G**, red hatched bar, *p *< 0.05), suggesting an association between hydrolase activity and hippocampal memory. *PrP-A53T-SNCA* mice displayed impaired cued memory responses at 4 months of age that were not further exacerbated by glucocerebrosidase inhibition (**H**). In open-field tests, there were no differences in ambulation or time spent in the center between *PrP-A53T-SNCA* and age-matched WT controls (**I, J**). All data are presented as the mean ± SEM. Bars with different letters are significantly different from each other (*p *< 0.05).

The motor abilities of WT and *PrP-A53T-SNCA* mice were initially evaluated at 8 weeks post-injection with CBE or saline using the pole test. Inhibition of glucocerebrosidase did not affect the performance of WT mice in the pole test. In contrast, *PrP-A53T-SNCA* mice showed a significant increase in the time required to turn from the upward position and initiate the descent (Fig. 2A; *PrP-A53T-SNCA* saline: 10 ± 4 s, *PrP-A53T-SNCA* CBE: 36 ± 9 s; *p *< 0.05) as well as the time required to descend the pole after turning (Fig. 2B; *PrP-A53T-SNCA* saline: 28 ± 5 s, *PrP-A53T-SNCA* CBE: 45 ± 7 s; *p *< 0.05). These results indicated that partial inhibition of glucocerebrosidase activity in *PrP-A53T-SNCA* mice affected their motor function.

The effects of CBE on motor skills were also evaluated after 8 weeks of treatment using the nest-building test, which assesses fine motor dexterity while imposing minimal stress on the mice. For this test, the animals were provided with 3 g of nesting squares (nestlet), and their nest scores as well as the amount of unused material were assessed after 16 h (34). CBE treatment had no effect on WT animals’ ability to make nests (Fig. 2C and D). *PrP-A53T-SNCA* mice injected with saline solution showed a slight reduction in their nest-building score, as previously reported (35). Inhibition of glucocerebrosidase in *PrP-A53T-SNCA* mice significantly decreased their nest-building scores (Fig. 2C; *PrP-A53T-SNCA* saline score: 3.9 ± 0.3, *PrP-A53T-SNCA* CBE score: 1.3 ± 0.2; *p *< 0.05). The deficiency in nest-building observed among *PrP-A53T-SNCA* mice and its exacerbation by CBE treatment was confirmed by quantifying the amount of unused nestlet material (Fig. 2D; *PrP-A53T-SNCA* saline: 0.9 ± 0.4 g, PrP-A53T-SNCA CBE: 2.7 ± 0.2 g; *p *< 0.05). Hind-limb splay, a qualitative measure of basic motor function (36), was similarly deficient in CBE-treated *PrP-A53T-SNCA* mice (Fig. 2E; *PrP-A53T-SNCA* saline score: 3.2 ± 0.2, *PrP-A53T-SNCA* CBE score: 1.8 ± 0.5; *p *< 0.05). Overall, the CBE-induced impairment in the performance of *PrP-A53T-SNCA* mice in the pole, nest-building and hind-limb splay tests indicates a relationship between reduced glucocerebrosidase activity and the development of fine motor deficits.

We also examined the effects of glucocerebrosidase activity on the gross motor function of the mice using the rotarod test. The performance of WT mice that had been treated with CBE for 9 weeks on the rotarod test was indistinguishable from the performance of mice in the saline control group (Fig. 2F). *PrP-A53T-SNCA* mice typically develop an overt motor phenotype starting at 8 months of age (31). However, at a younger age, this mouse model exhibited improved motor performance on the rotarod test (Fig. 2F; WT saline: 167 ± 17 s, *PrP-A53T-SNCA* saline: 251 ± 17 s; *p *< 0.05); this is similar to previous reports of 2- and 6-month-old mice (31,35). CBE-induced inhibition of glucocerebrosidase activity had no effect on the latency of *PrP-A53T-SNCA* mice to fall from the rod (Fig. 2F; *PrP-A53T-SNCA* saline: 251 ± 17 s, *PrP-A53T-SNCA* CBE: 258 ± 14 s; *p *> 0.05), suggesting that partial enzyme inhibition at this early time point did not affect this motor behavior.

Next, we evaluated the effect of glucocerebrosidase inhibition on cognition using a cued and contextual fear-conditioning paradigm. Animals treated for 10 weeks with CBE or saline were first exposed to a conditioned (tone) stimulus followed by an unconditioned (shock) stimulus. After 24 h, context-specific and tone-related freezing behaviors were measured consecutively (see Materials and Methods section). The contextual memory assessment showed no differences between saline-treated WT and *PrP-A53T-SNCA* mice (Fig. 2G). Remarkably, glucocerebrosidase inhibition significantly worsened the performance of *PrP-A53T-SNCA* mice in the contextual memory task (Fig. 2G; *PrP-A53T-SNCA* saline: 39 ± 6% freezing, *PrP-A53T-SNCA* CBE: 20 ± 6% freezing; *p *< 0.05). This observation confirmed the importance of glucocerebrosidase activity in hippocampal memory as suggested previously in a Gaucher-related synucleinopathy mouse model (26). On the other hand, the cued memory task was significantly reduced in saline-treated *PrP-A53T-SNCA* mice compared with control WT mice (Fig. 2H; WT saline: 34 ± 4 s, *PrP-A53T-SNCA* saline: 14 ± 3 s; *p *< 0.05), and this task was not further affected by CBE inhibition. These data suggest the presence of a previously unrecognized amygdala-dependent memory deficit in this transgenic model and indicate that the level of glucocerebrosidase inhibition attained in these studies was not sufficient to affect the cued memory deficits. Importantly, none of the deficits observed in saline or CBE-injected mice were related to a loss of ambulatory activity or an increase in anxiety, a judgment based on similar performance to control mice in an open-field test (Fig. 2I and J). Taken together, these results suggest that a reduction in glucocerebrosidase activity contributes to the pathophysiology of *GBA1*-related synucleinopathies and plays a role in the development of specific motor and cognitive deficits.

### Lowering glucocerebrosidase activity further exacerbates transcriptional aberrations in *PrP-A53T-SNCA* animals

To gain further insights into the molecular mechanisms underlying the increase in behavioral dysfunction associated with decreased glucocerebrosidase activity in *PrP-A53T-SNCA* mice, we compared the gene expression profiles in the striata of WT and *PrP-A53T-SNCA* mice treated with CBE or vehicle. We focused our assessment on genes that have been associated with the development of Gaucher disease, such as genes implicated in the metabolism of glucosylceramide as well as inflammation- and RIPK-mediated responses (Table 1) (27,37). We also analyzed genes that are associated with neuronal function, the dopaminergic pathway, synaptic transmission, calcium-dependent responses and the synaptic vesicle fusion complex SNARE (38–40).

**Table 1.** Effects of partial glucocerebrosidase inhibition on gene expression in the striata of WT and *PrP-A53T-SNCA* mice

Category	Protein	Gene name	*WT* Saline (mean ± SEM)	*WT* CBE^a^ (mean ± SEM)	*PrP-A53T-SNCA* Saline (mean ± SEM)	*PrP-A53T-SNCA* CBE (mean ± SEM)	Entrez ID	Primer probe set (Catalog #)
Ca-related	Calbindin	*Calb1*	1.00 ± 0.07	0.91 ± 0.10	1.02 ± 0.08	1.12 ± 0.08	12307	Mm00486647_m1
	Calbindin 2	*Calb2*	1.00 ± 0.37	0.88 ± 0.17	0.56 ± 0.12	0.40 ± 0.06^c^	12308	Mm00801461_m1
	Calregulin	*Calr*	1.00 ± 0.09	0.99 ± 0.10	0.86 ± 0.09	1.01 ± 0.06	12317	Mm00482936_m1
	Calcium/calmodulin-dependent protein kinase I	*Camk1*	1.00 ± 0.13	1.20 ± 0.11	0.95 ± 0.25	0.60 ± 0.12^b^^,^^c^	52163	Mm00519436_m1
	Calcium/calmodulin-dependent protein kinase II	*Camk2a*	1.00 ± 0.07	0.93 ± 0.08	0.91 ± 0.09	0.85 ± 0.10	12322	Mm00437967_m1
	Serine/threonine-protein phosphatase 2B	*Ppp3ca*	1.00 ± 0.02	1.07 ± 0.08	1.10 ± 0.04	1.27 ± 0.06^b^	19055	Mm01317678_m1
	Ryanodine receptor 3	*Ryr3*	1.00 ± 0.08	0.87 ± 0.07	0.87 ± 0.07	1.24 ± 0.10^c^	20192	Mm01328421_m1
Control	β-actin	*Actb*	1.00 ± 0.03	1.04 ± 0.10	0.93 ± 0.08	0.97 ± 0.06	11461	Mm00607939_s1
	GADPH	*Gadph*	1.00 ± 0.07	0.94 ± 0.07	0.87 ± 0.03	0.84 ± 0.07	14433	Mm99999915_g1
	HPRT	*Hprt*	1.00 ± 0.07	0.99 ± 0.11	0.85 ± 0.10	0.98 ± 0.13	15452	Mm01545399_m1
	–	*18S MOUSE*	1.00 ± 0.07	1.05 ± 0.10	0.87 ± 0.17	1.08 ± 0.07	19791	Mm03928990_g1
Dopaminergic	TH	*Th*	1.00 ± 0.14	1.45 ± 0.18	0.98 ± 0.04^c^	1.39 ± 0.17	21823	Mm00447557_m1
	BDNF	*Bdnf*	1.00 ± 0.15	1.14 ± 0.25	0.58 ± 0.09^b^^,c^	0.33 ± 0.06^b^^,c^	12064	Mm01334042_m1
	BDNF	*Bdnf*	1.00 ± 0.20	0.86 ± 0.20	0.40 ± 0.09^b^^,c^	0.21 ± 0.06^b^^,c^	12064	Mm04230607_s1
	CB1-Cannabinoid receptor 1	*Cnr1*	1.00 ± 0.05	0.99 ± 0.09	0.94 ± 0.07	1.07 ± 0.16	12801	Mm00432621_s1
	Dopamine receptor D2	*Drd2*	1.00 ± 0.07	1.07 ± 0.11	1.25 ± 0.11	1.28 ± 0.12	13489	Mm00438545_m1
	Preproenkephalin	*Penk*	1.00 ± 0.10	0.90 ± 0.09	1.08 ± 0.08	1.06 ± 0.05	18619	Mm01212875_m1
	DARPP-32	*Ppp1r1b*	1.00 ± 0.08	1.07 ± 0.10	1.15 ± 0.16	1.51 ± 0.10^b^^,c^	19049	Mm00454892_m1
	Vesicular monoamine transporter 2 (VMAT2)	*Slc18a2*	1.00 ± 0.55	0.59 ± 0.11	0.55 ± 0.08	0.43 ± 0.08	214084	Mm00553062_m1
	DAT	*Slc6a3*	1.00 ± 0.20	0.88 ± 0.22	1.10 ± 0.52	0.79 ± 0.30	13162	Mm00438396_m1
	Glutamate decarboxylase 1	*Gad1*	1.00 ± 0.10	1.11 ± 0.11	0.91 ± 0.04	1.14 ± 0.09	14415	Mm00725661_s1
	NOS1	*Nos1*	1.00 ± 0.07	0.92 ± 0.02	0.91 ± 0.09	0.97 ± 0.02	18125	Mm00435175_m1
	NPY	*Npy*	1.00 ± 0.07	1.12 ± 0.06	0.97 ± 0.07	0.84 ± 0.04^c^	109648	Mm03048253_m1
	Parvalbumin	*Pvalb*	1.00 ± 0.13	1.54 ± 0.41	0.74 ± 0.19	0.96 ± 0.18	19293	Mm00443100_m1
	Dopamine receptor D1	*Drd1a*	1.00 ± 0.05	1.16 ± 0.11	1.03 ± 0.14	1.40 ± 0.07^b^	13488	Mm01353211_m1
	Prodynorphin	*Pdyn*	1.00 ± 0.12	0.89 ± 0.14	0.92 ± 0.04	0.88 ± 0.14	18610	Mm00457572_m1
	Tac1	*Tac1*	1.00 ± 0.13	0.93 ± 0.10	0.93 ± 0.05	1.08 ± 0.10	21333	Mm01166996_m1
Inflammation	IBA1	*Aif1*	1.00 ± 0.09	0.76 ± 0.14	1.17 ± 0.31	1.11 ± 0.33	11629	Mm00479862_g1
	GFAP	*Gfap*	1.00 ± 0.09	1.13 ± 0.09	1.48 ± 0.23	4.00 ± 1.15^b^^,c^	14580	Mm01253033_m1
GlcCer pathway	GBA1	*Gba*	1.00 ± 0.07	1.04 ± 0.08	0.90 ± 0.07	0.86 ± 0.07	14466	Mm00484700_m1
	GBA2	*Gba2*	1.00 ± 0.16	0.78 ± 0.10	0.80 ± 0.10	0.91 ± 0.06	230101	Mm00554547_m1
	Saposin	*Psap*	1.00 ± 0.06	1.07 ± 0.07	0.86 ± 0.10	0.98 ± 0.10	19156	Mm00478327_m1
	LIMP-2	*Scarb2*	1.00 ± 0.15	1.15 ± 0.26	1.05 ± 0.37	0.87 ± 0.08	12492	Mm00446978_m1
	GluCer Synthase	*Ugcg*	1.00 ± 0.08	0.94 ± 0.06	0.85 ± 0.05	0.85 ± 0.04	22234	Mm00495925_m1
Lysosomal biogenesis	TFEB	*Tfeb*	1.00 ± 0.08	0.98 ± 0.10	0.84 ± 0.13	1.03 ± 0.10	21425	Mm00448968_m1
RIPK pathway	CASP8	*Cflar*	1.00 ± 0.04	1.07 ± 0.08	0.98 ± 0.10	1.14 ± 0.17	12633	Mm01255578_m1
	RIPK1	*Ripk1*	1.00 ± 0.14	1.19 ± 0.23	1.22 ± 0.22	1.14 ± 0.26	19766	Mm00436354_m1
	RIPK3	*Ripk3*	1.00 ± 0.41	1.53 ± 0.23	2.19 ± 0.32^b^	3.65 ± 1.13^b^	56532	Mm01319233_g1
SNARE	Alpha-soluble NSF attachment protein	*Napa*	1.00 ± 0.04	1.02 ± 0.06	0.97 ± 0.05	0.97 ± 0.03	108124	Mm00445948_m1
	Synaptosomal-associated protein 25	*Snap25*	1.00 ± 0.08	0.97 ± 0.11	0.87 ± 0.06	0.80 ± 0.08	20614	Mm00456921_m1
	Syntaxin16	*Stx16*	1.00 ± 0.07	0.88 ± 0.05	0.80 ± 0.07	0.87 ± 0.03	228960	Mm00660009_m1
	Syntaxin 1a	*Stx1a*	1.00 ± 0.15	0.82 ± 0.18	0.58 ± 0.07^b^	0.37 ± 0.04^b^^,c^	20907	Mm00444008_m1
	Syntaxin6	*Stx6*	1.00 ± 0.14	0.97 ± 0.10	0.87 ± 0.05	1.05 ± 0.13	58244	Mm01295595_m1
	Synaptotagmin VII	*Syt7*	1.00 ± 0.06	0.87 ± 0.11	0.69 ± 0.11^b^	0.75 ± 0.08^b^	54525	Mm01199320_s1
	Synaptobrevin 2 (VAMP2)	*Vamp2*	1.00 ± 0.26	0.92 ± 0.18	0.92 ± 0.11	0.67 ± 0.07	22318	Mm01325243_m1
Synaptic	PSD95	*Dlg4*	1.00 ± 0.10	0.90 ± 0.06	0.97 ± 0.04	0.94 ± 0.08	13385	Mm00492193_m1
	mGluR1	*Grm1*	1.00 ± 0.04	0.99 ± 0.06	1.09 ± 0.12	1.10 ± 0.06	14816	Mm00810231_s1
	mGluR5	*Grm5*	1.00 ± 0.07	0.99 ± 0.10	1.02 ± 0.05	1.07 ± 0.03	108071	Mm00690332_m1
	Huntingtin-interacting protein 1	*Hip1*	1.00 ± 0.05	0.88 ± 0.05	0.85 ± 0.08	0.91 ± 0.05	215114	Mm00524503_m1
	Kinesin heavy chain	*Kif5b*	1.00 ± 0.09	1.17 ± 0.13	1.10 ± 0.11	1.29 ± 0.09^b^	16573	Mm00515276_m1
	Myosin Vb	*Myo5b*	1.00 ± 0.12	1.14 ± 0.08	1.07 ± 0.07	1.26 ± 0.14	17919	Mm00485360_m1
	NeuN	*Rbfox3*	1.00 ± 0.15	0.88 ± 0.05	1.09 ± 0.15	1.15 ± 0.21	52897	Mm01248771_m1
	Synapsin I	*Syn1*	1.00 ± 0.03	0.92 ± 0.03	0.85 ± 0.04^b^	0.62 ± 0.06^b,c^	20964	Mm00449772_m1
	Synapsin II	*Syn2*	1.00 ± 0.04	0.81 ± 0.09	0.75 ± 0.14	0.56 ± 0.04^b^^,c^	20965	Mm00449780_m1
	Synaptophysin	*Syp*	1.00 ± 0.13	0.82 ± 0.10	0.67 ± 0.05^b^	0.53 ± 0.04^b^^,c^	20977	Mm00436850_m1

Mice were injected with saline or CBE (100 mg/kg, three times per week, 10 weeks), and total striatal mRNA expression was assessed by qRT-PCR as described in the Materials and Methods section. The expression of these striatal genes was not significantly affected by genotype in 4-month-old saline-treated animals. CBE-mediated glucocerebrosidase inhibition elicited some specific transcriptional responses. Some genes were particularly affected by glucocerebrosidase inhibition in both genotypes, including dopaminergic genes, synaptic genes, SNARE-associated genes and *Ripk3*. The number of altered transcripts was increased by overexpression of mutant α-synuclein and included a larger subset of genes as well as transcripts involved in calcium-dependent responses and astrocyte activation. The data are presented as the mean ± SEM (ANOVA followed by Newman–Keuls, *n* ≥ 5 per group).

^a^WT-saline was not different from *PrP-A53T-SNCA-*saline.

^b^Significantly different from WT-saline (*p *< 0.05).

^c^Significantly different from *PrP-A53T-SNCA-*saline (*p *< 0.05).

Analysis of the transcripts of 4-month-old WT and *PrP-A53T-SNCA* mice (Table 1) showed that expression of their striatal genes was not significantly different at this age. However, partial inhibition of glucocerebrosidase activity with CBE elicited some changes in their transcriptional profiles. Transcripts that were particularly affected by glucocerebrosidase inhibition in both WT and *PrP-A53T-SNCA* animals included genes in the dopaminergic, synaptic and SNARE group (i.e., *Bdnf*, *Stx1a, Syt7, Syn1* and *Syp*) as well as *Ripk3*. The number of altered transcripts and their levels were greater in mice overexpressing mutant α-synuclein, including transcripts involved in calcium-dependent responses and astrocyte activation (i.e. *Calb2, Camk1*, *Ryr3, Ppp1r1b, Npy* and *Gfap*; Table 1). Notably, genes involved in the metabolism of glucosylceramide were not modified by partial inhibition of glucocerebrosidase (i.e. *Gba1, Gba2, Psap, Scarb2, Tfeb, Ugcg*). Taken together, these results suggest a role for glucocerebrosidase in normal neuronal function and indicate that a reduction of its activity could lead to enhancement of the deleterious effects of α-synuclein accumulation.

### Administration of AAV1-GBA1 into the striatum of female *Thy1-SNCA* mice increases glucocerebrosidase activity and reduces α-synuclein pathology

**Figure 3.**
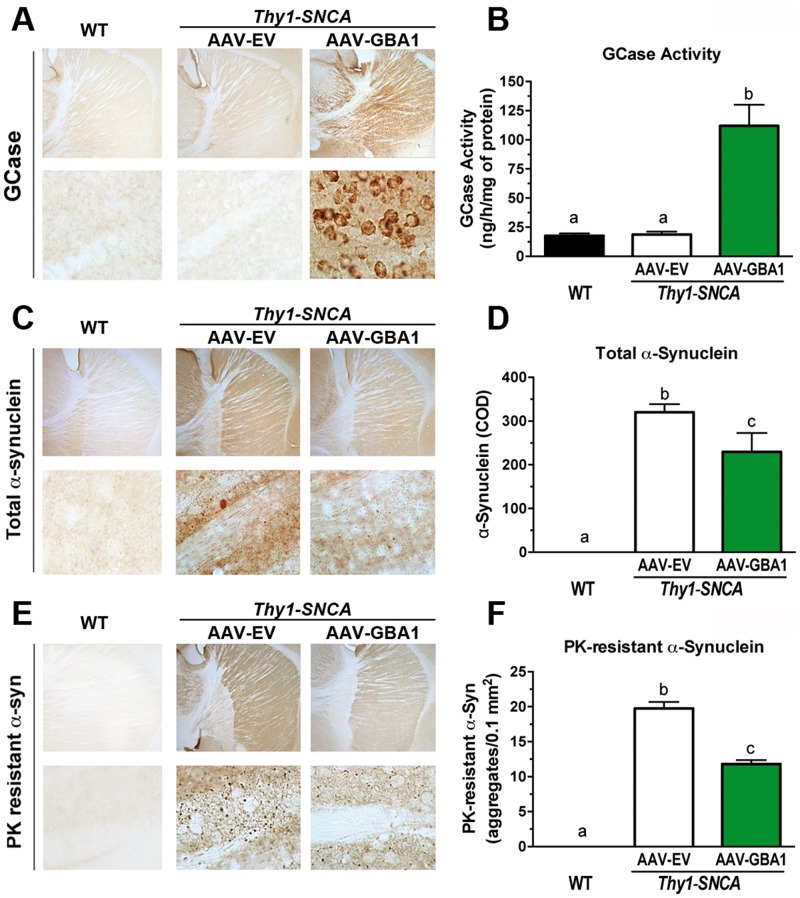
Administration of AAV1-GBA1 into the striatum of *Thy1-SNCA* transgenic mice reduces the levels of α-synuclein in the CNS. Two-month-old female *Thy1-SNCA* mice were subjected to bilateral striatal injections of 1.8e10 gc/site of either AAV1-EV (*n* = 16, white columns) or AAV1-GBA1 (*n* = 16, green columns). Uninjected, age-matched WT mice (WT; *n* = 16, black columns) were used as healthy controls. Tissues were collected for biochemical and pathological analysis at 7 months post-injection. Immunostaining for glucocerebrosidase showed robust striatal expression in AAV1-GBA1-injected *Thy1-SNCA* mice. Mice treated with a control virus (AAV1-EV) showed a low level of staining in the CNS that was similar in intensity to that in untreated WT mice. Representative pictures are shown in (**A**). Robust glucocerebrosidase activity was observed in AAV1-GBA1-injected striata (**B**, 6-fold greater than the AAV1-EV controls, *n* = 8 per group, *p *< 0.05). Increased glucocerebrosidase activity in the CNS of *Thy1-SNCA* mice reduced the levels of total and proteinase K-resistant α-synuclein levels. Representative photomicrographs of brain sections from the striata of WT and *Thy1-SNCA* mice immunostained with an anti-α-synuclein antibody without (**C**) and with (**E**) proteinase K pretreatment. Quantification of the α-synuclein signal indicated a significant reduction in total and proteinase K-resistant α-synuclein in the AAV1-GBA1 treated group. Graphs represent striatal quantification of total α-synuclein (**D**, *n* = 16 per group) and proteinase K-resistant α-synuclein (**F**, *n* = 16 per group). The results are presented as the mean ± SEM. Bars marked with different letters are significantly different from each other (*p *< 0.05).

We previously demonstrated that augmenting glucocerebrosidase activity in the CNS can reduce α-synuclein levels and correct the behavioral impairments observed in a mouse model of Gaucher disease (26,30). Overexpression of glucocerebrosidase in the CNS of the *PrP-A53T-SNCA* mice reduced soluble α-synuclein levels but did not affect their motor or survival endpoints, presumably because of the severity of the disease in this animal model (30). Here, we sought to determine if reconstituting the CNS with recombinant glucocerebrosidase could ameliorate the functional and pathological aberrations in a mouse model of milder synucleinopathy. *Thy1-SNCA* transgenic mice express lower levels of α-synuclein than *PrP-A53T-SNCA* mice and do not show evidence of motor neuron degeneration (41). For our studies, we elected to use only female *Thy1-SNCA* mice, as they express a lower level of α-synuclein than their male littermates (41,42). A recombinant adeno-associated viral vector (serotype 1) encoding human glucocerebrosidase (AAV1-GBA1) was administered bilaterally into the striatum of 2-month-old *Thy1-SNCA* transgenic females. A control group received an empty AAV vector lacking a transgene (AAV1-EV). Immunohistochemical examination of the CNS of *Thy1-SNCA* mice at 7 months post-treatment revealed abundant and widespread staining of human glucocerebrosidase (Fig. 3A). Measurement of glucocerebrosidase activity showed highly elevated levels of the enzyme (∼6-fold higher than controls) within the striatum following administration of AAV1-GBA1 (Fig. 3B; *Thy1-SNCA* AAV1-GBA1: 112 ± 18 ng/h/mg of protein, *p *< 0.05 versus *Thy1-SNCA* AAV1-EV).

Brains from PD patients with no mutations in *GBA1* can exhibit decreased glucocerebrosidase activity (32), presumably due to accumulation of α-synuclein (43,44). To explore this phenomenon further, we measured glucocerebrosidase levels in the CNS of mice accumulating progressively higher levels of α-synuclein. Glucocerebrosidase activity in the brains of *Thy1-SNCA* mice was not different from age-matched WT controls (Fig. 3B; WT: 18 ± 2 ng/h/mg of protein; *Thy1-SNCA* AAV1-EV: 19 ± 2 ng/h/mg of protein). This finding is consistent with a previous report showing that the glucocerebrosidase activity of mice showing a modest increase (<2-fold) in α-synuclein levels is not affected (29). In contrast, brains from *PrP-SNCA* mice, which showed higher levels of α-synuclein (45), had decreased levels of glucocerebrosidase (Fig. 1A), suggesting that high levels of α-synuclein can modulate glucocerebrosidase activity. Hence, there is a correlation between higher α-synuclein levels and reduced glucocerebrosidase activity in the CNS.

Accumulation of intraneuronal α-synuclein inclusions is a hallmark of PD (46). *Thy1-SNCA* mice exhibit progressive and marked accumulation of proteinase K-resistant α-synuclein aggregates in the CNS (41). Immunohistochemical analysis of the striata of AAV1-GBA1- and AAV1-EV-treated *Thy1-SNCA* mice at 7 months post-injection showed that increasing glucocerebrosidase activity significantly reduced the levels of total human α-synuclein (Fig. 3C and D, 28% reduction in AAV1-GBA1-injected mice compared with the control group). Increased glucocerebrosidase activity in the striatum of *Thy1-SNCA* mice also resulted in a reduction in the proteinase K-resistant fraction of α-synuclein aggregates (Fig. 3E and F, 40% reduction in AAV1-GBA1-injected mice compared with the control group). These results are in agreement with our previous findings in a Gaucher-associated mouse model of synucleinopathy (and support the contention that augmenting glucocerebrosidase activity can restore α-synuclein homeostasis and the pathological deposition of α-synuclein in animals carrying WT *Gba1* alleles).

### Administration of AAV1-GBA1 into the striatum prevents hyperactivity in *Thy1-SNCA* female mice

**Figure 4.**
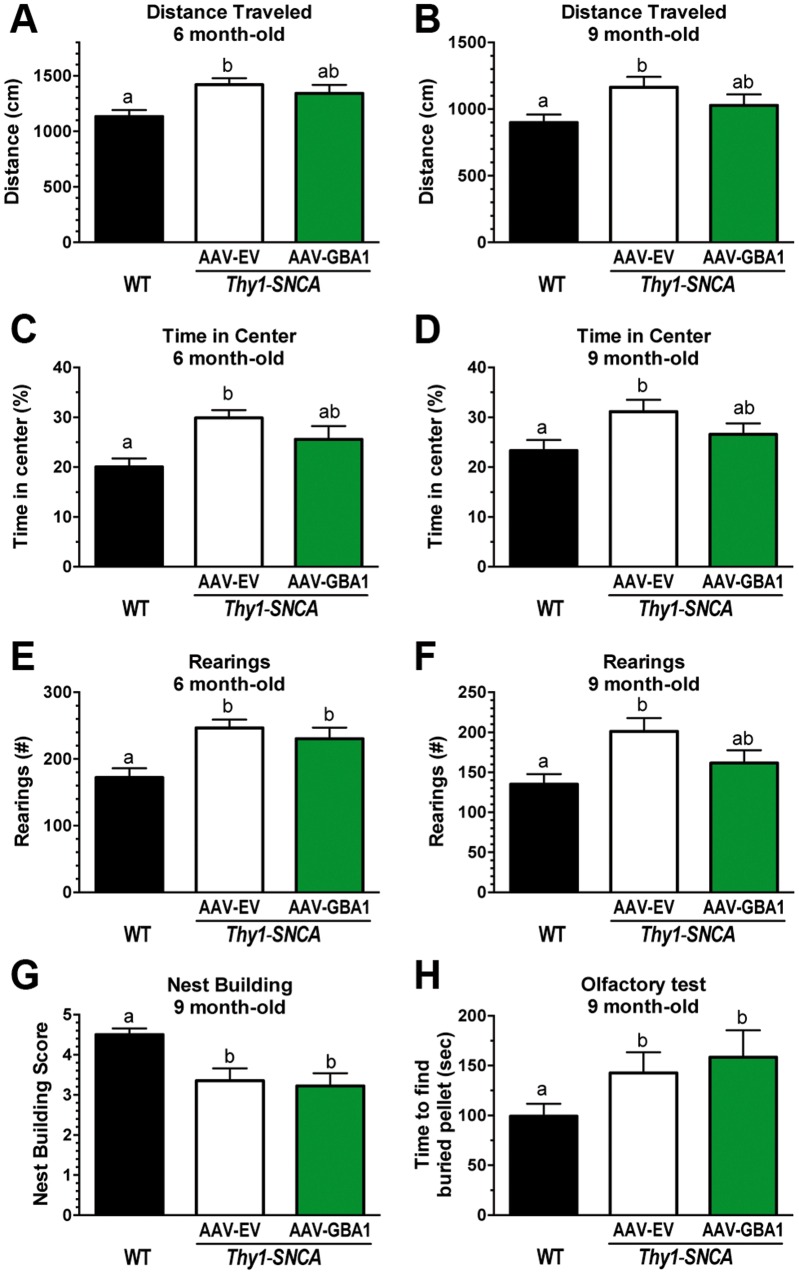
Administration of AAV1-GBA1 into the striatum of *Thy1-SNCA* mice prevents the development of behavioral abnormalities. Two-month-old female *Thy1-SNCA* mice were subjected to bilateral striatal injections of either AAV1-EV (*n* = 16, white columns) or AAV1-GBA1 (*n* = 16, green columns). Uninjected age-matched WT mice (*n* = 16, black columns) were used as a positive control. Animals were subjected to the open field test at 4 and 7 months post-injection. Treatment with AAV1-GBA1 prevented the development of hyperactivity and decreased the time the animals spent in the center of the arena at both time points (**A–D**). AAV1-GBA1-treated *Thy1-SNCA* mice also displayed a reduced number of investigational rearings at 9 months of age (**E, F**). However, increasing striatal glucocerebrosidase activity did not improve the nest-building scores or the olfactory function of *Thy1-SNCA* mice at 9 months of age (**G, H**), suggesting that localized enzyme supplementation could not overcome the generalized pathology in this mouse model. All data are presented as the mean ± SEM. Bars with different letters are significantly different from each other (*p *< 0.05).

The locomotor activity of *Thy1-SNCA* mice was evaluated at 6 and 9 months of age (i.e. 4 and 7 months post-surgery) using the open field exploration test. Female *Thy1-SNCA* mice presented with hyperactivity, increased risk behavior and more investigational rearing activity compared with WT animals. This early phenotype is hypothesized to be due to dysregulation of the striatal dopaminergic system (42,47,48). Treatment of *Thy1-SNCA* mice with AAV1-GBA1 prevented the development of hyperactivity and impulsive behavior at both the 6- and 9-month time points as measured by the total distance traveled and time spent in the center of the open field (Fig. 4A–D). The AAV1-GBA1-mediated increase in glucocerebrosidase activity in the CNS also reduced the number of vertical investigations at the later time point (Fig. 4E and F; 9-month time point: WT: 135 ± 13 rears; *Thy1-SNCA* AAV1-EV: 201 ± 17 rears, *p *< 0.05 versus WT; *Thy1-SNCA* AAV1-GBA1 162 ± 16 rears, *p *> 0.05 versus WT). Taken together, these data indicate that augmenting glucocerebrosidase in the striatum of female *Thy1-SNCA* mice could confer a protective benefit against the development of functional deficits.

The ability of higher striatal levels of glucocerebrosidase to ameliorate the nest-building and olfactory deficits in *Thy1-SNCA* mice was also evaluated. *Thy1-SNCA* female mice exhibited these aberrant behaviors beginning at nine months of age (Fig. 4G and H; nest-building score: WT: 4.5 ± 0.2; *Thy1-SNCA* AAV1-EV: 3.4 ± 0.3, *p *< 0.05; time to find a buried pellet: WT: 99 ± 12 s; *Thy1-SNCA* AAV1-EV: 143 ± 21 s, *p *< 0.05). Treatment of *Thy1-SNCA* animals with AAV1-GBA1 did not modify their nest-building score or time to find a buried pellet compared with the AAV1-EV-treated control group (Fig. 4G and H; nest-building score: *Thy1-SNCA* AAV1-GBA1: 3.2 ± 0.3; time to find a buried pellet: *Thy1-SNCA* AAV1-GBA1: 158 ± 27 s). These results indicated that widespread expression of the glycosidase throughout the CNS might be necessary to correct non-striatal deficits.

### Administration of AAV1-GBA1 into the striatum prevents dopaminergic deficits in *Thy1-SNCA* mice

**Figure 5.**
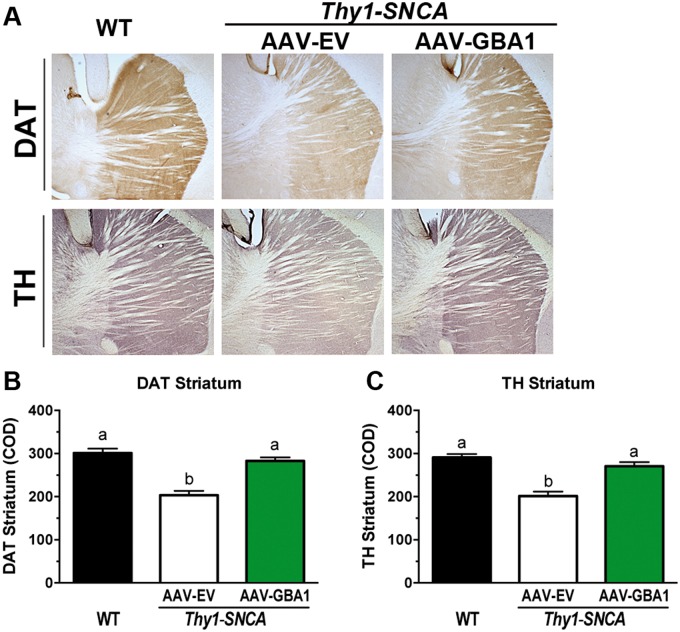
Administration of AAV1-GBA1 into the striatum of *Thy1-SNCA* mice prevents loss of dopaminergic markers. Female *Thy1-SNCA* mice were subjected to bilateral striatal injections of either AAV1-EV (*n* = 16, white columns) or AAV1-GBA1 (*n* = 16, green columns) at 2 months of age, as outlined in the legend of Figure 3. Tissues were collected for analysis at 9 months of age. Sections were stained with antibodies against the DAT or TH. (**A**) Images show representative photomicrographs of DAT and TH immunostaining. Nine-month-old AAV1-EV-treated *Thy1-SNCA* mice displayed a significant reduction in DAT (**B**) and TH (**C**) immunostaining in the striatum. This decrease was prevented by overexpression of glucocerebrosidase (green columns). Graphs represent quantification of total DAT (B) and TH (C) immunostaining in the striatum. The results are presented as the mean ± SEM. Bars with different letters are significantly different from each other (*p *< 0.05).

PD is characterized by progressive degeneration of the dopaminergic terminals, which precedes the neuronal cell loss (49). This feature is recapitulated in the *Thy1-SNCA* mouse model, which reveals a progressive process of terminal loss without decline in the number of tyrosine hydroxylase (TH)-positive dopaminergic neurons in the substantia nigra (41,42). We sought to evaluate whether striatal glucocerebrosidase augmentation through AAV-mediated gene transfer could protect the dopaminergic terminal degeneration in *Thy1-SNCA* mice. The dopaminergic system was evaluated by immunohistochemical staining for the dopamine active transporter (DAT) and TH. Transport of dopamine mediated by DAT is the primary mechanism by which dopamine is cleared from synapses (50). TH catalyzes the rate-limiting step in the synthesis of catecholamines, including dopamine (51). Morphometric analysis of the brain tissues of female *Thy1-SNCA* mice at 7 months post-injection with the control AAV1-EV vector revealed a significant reduction in the DAT signal (Fig. 5A and B; WT: 301 ± 10 COD; *Thy1-SNCA* AAV1-EV: 203 ± 10 COD; *p *< 0.05). *Thy1-SNCA* mice administered AAV1-GBA1 (at 2 months of age) showed no reduction in striatal DAT immunoreactivity (Fig. 5A and B; *Thy1-SNCA* AAV1-GBA1: 283 ± 8 COD, *p *< 0.05 versus *Thy1-SNCA* AAV1-EV). Similar results were observed for the catecholamine synthetic enzyme; *Thy1-SNCA* animals treated with control AAV1-EV but not AAV1-GBA1 displayed a significant decrease in TH optical density (Fig. 5A and C; WT: 291 ± 8 COD; *Thy1-SNCA* AAV1-EV: 202 ± 10 COD, *p *< 0.05; Thy1*-SNCA* AAV1-GBA1: 270 ± 10 COD, *p *> 0.05).

To confirm the salutary effects of increased glucocerebrosidase activity in the striatum of *Thy1-SNCA* mice, we measured the mRNA levels of several dopaminergic and neuronal targets using the same target genes described in Table 1. Quantitative RT-PCR analyses showed that only two of these transcripts were significantly altered in *Thy1-SNCA* controls compared with WT controls (i.e. dopamine receptor D2 and DAT). Importantly, the levels of these two transcripts were normalized in *Thy1-SNCA* mice injected with AAV1-GBA1 (*Drd2*: *Thy1-SNCA* AAV1-EV: 1.30 ± 0.09, *p *< 0.05 versus WT; *Thy1-SNCA* AAV1-GBA1: 1.14 ± 0.22, *p *> 0.05 versus WT; *Slc6a3*: *Thy1-SNCA* AAV1-EV: 1.67 ± 0.12, *p *< 0.05 versus WT; *Thy1-SNCA* AAV1-GBA1: 1.02 ± 0.12, *p *> 0.05 versus WT), confirming the protective effect of glucocerebrosidase on dopaminergic terminals. Taken together, these results indicate that augmenting the levels of glucocerebrosidase in the CNS of *Thy1-SNCA* mice could prevent age-dependent dopaminergic deficits.

## Discussion

Following the initial discovery that mutations in *GBA1* are a risk factor for developing PD and DLB (2,52), findings from several independent studies have supported a direct role for glucocerebrosidase in the pathogenesis of these devastating diseases (4,16). For example, a decrease in glucocerebrosidase activity and/or the presence of a mutant enzyme can purportedly induce an increase in CNS α-synuclein/ubiquitin aggregates (24– 26,29,43,53,54). Conversely, increasing glucocerebrosidase levels in the CNS can abate the pathological accumulation of α-synuclein in the hippocampus of mouse models of Gaucher-related synucleinopathy (26,30). Notably, PD patients without *GBA1* mutations can exhibit lower levels of glucocerebrosidase enzyme in blood and CNS, further implicating this lysosomal enzyme in the pathogenesis of PD (32,55). This observation suggests that glucocerebrosidase activity (regardless of the presence of a mutant protein) may play a significant role in the disease. Here, we further investigated the potential role of glucocerebrosidase activity in modulating disease progression in two mouse models of synucleinopathy that harbored wild-type *Gba1* alleles. This study supports a role for glucocerebrosidase activity in α-synuclein processing that is independent of mutations in *GBA1.* We showed that a reduction in this hydrolase activity led to the development of motor and non-motor morbidities. Increasing the enzymatic activity attenuated and delayed disease progression. Collectively, these data indicate that glucocerebrosidase augmentation in the CNS may be a potential therapeutic approach for diseases associated with α-synuclein misprocessing such as PD and DLB.

Genetic variations in *GBA1* are emerging as a significant feature impacting the natural history of PD. Patients who harbor *GBA1* mutations present a higher prevalence and severity of bradykinesia, motor complications, cognitive decline and anxiety (6,11,15). This study demonstrated that a partial reduction in glucocerebrosidase activity recapitulated the negative impact of *GBA1* mutations on specific behaviors. Exposure of the A53T-α-synuclein mouse model of synucleinopathy to a brain-penetrant inhibitor of glucocerebrosidase (CBE) exacerbated the animals’ fine motor deficits and decreased their hippocampal memory function. These behavioral abnormalities were correlated with an increase in the accumulation of α-synuclein and glucosylsphingosine in the CNS. A similar increase in both α-synuclein and lipids has been reported in brain tissues and iPSC-derived neurons from PD patients carrying heterozygous *GBA1* mutations (33,56). Decreased glucocerebrosidase activity in Gaucher models cause changes the glycosphingolipid membrane composition of lysosomes and associated organelles. This accumulation can affect membrane trafficking along the endocytic pathway as well as the lysosomal degradative function (57–59). Importantly, even small changes in the lipid membrane composition can affect α-synuclein homeostasis and trigger the formation of pathological amyloid fibrils (60,61), hence interfering with α-synuclein normal functions in vesicle trafficking and synaptic plasticity of neurons. At face value, these results would suggest that the aberrant accumulation of lipids and α-synuclein are downstream effects of PD and that strategies to reduce the accumulation of these entities may be therapeutic. Here, we showed that enzyme augmentation therapy represents one such approach, but strategies that reduce the production of these entities should also be considered.

Pharmacological inhibition of glucocerebrosidase activity *per se* was not sufficient to affect certain behaviors in *PrP-A53T-SNCA* mice (e.g. rotarod and ambulatory ability). Correspondingly, a recent study showed that a double transgenic mouse expressing the L444P variant of *Gba1* and four copies of human α-synuclein presented rotarod deficits only after 14 months of age (29). Hence, it is possible that prolonged inhibition of the enzymatic activity may be required to impact specific circuits.

A battery of transcripts associated with glucocerebroside metabolism or with normal neuronal function were examined in the striatum of WT and *PrP-A53T-SNCA* transgenic mice following treatment with saline or CBE. Partial inhibition of glucocerebrosidase activity in WT animals promoted mild dysregulation of specific genes involved in synaptic transmission and vesicle trafficking (e.g. *Bdnf*, *Stx1a, Syt7, Syn1* and *Syp*). The CBE-mediated dysregulation was markedly exacerbated in mice overexpressing α-synuclein and included genes involved in calcium-regulated responses and astroglial inflammation (e.g. *Calb2, Camk1*, *Ryr3, Ppp1r1b, Npy* and *Gfap*). This synergistic deleterious effects of inhibiting glucocerebrosidase in the context of high levels of α-synuclein provide further support for the previously suggested pathogenic feedback loop between glucocerebrosidase and α-synuclein (43). The results indicated that decreasing glucocerebrosidase activity could further compromise specific neuronal functions, which may contribute to the earlier and greater impairments in neuronal function, as noted in PD patients carrying *GBA1* mutations (6,11,15,62). Cognitive impairment occurs more frequently in Gaucher disease patients and carriers than in controls (9,10). Brain-derived neurotrophic factor (BDNF) is an activity-dependent secreted protein that has a critical role in the organization of neuronal networks and synaptic plasticity (63). The BDNF mRNA level was reduced by glucocerebrosidase inhibition and was further decreased in the α-synuclein transgenic animals treated with CBE. Interestingly, these mice displayed the lowest performance on the contextual memory test. A recent study showed that treatment of Gaucher patients with recombinant glucocerebrosidase increased serum BDNF levels (64). However, as the recombinant enzyme does not transverse the blood–brain barrier, the mechanisms and clinical relevance of these findings still need to be addressed.

Modulation of the glucocerebrosidase metabolic pathway as a therapeutic strategy for synucleinopathies has been partially validated through human genetics and preclinical studies (16). Our previous studies demonstrated that AAV-mediated expression of glucocerebrosidase in the CNS of mice with both pre- and post-symptomatic Gaucher disease was efficacious at reducing α-synuclein accumulation and reversing the associated cognitive impairment (26). The present report extends this observation to include murine models of synucleinopathies that harbor WT alleles of *Gba1* (e.g. *Thy1-SNCA* transgenic mice). Reconstituting the CNS with exogenous enzyme was associated with a measurable reduction in the accumulation of α-synuclein aggregates. Augmenting glucocerebrosidase activity in the striata of *Thy1-SNCA* mice prevented the development of dopaminergic aberrations, as evidenced by normalization of the protein and mRNA profiles and improvements in the behavioral function of the mice. Hence, increasing glucocerebrosidase activity in the CNS of this mouse model of PD restored the cellular capacity to degrade α-synuclein and prevented the development of α-synuclein-dependent deficits (65,66). Augmenting glucocerebrosidase activity in the CNS via administration of the recombinant enzyme, gene transfer vectors expressing the lysosomal enzyme, or small-molecule activators of hydrolase may represent potential strategies to slow disease progression in PD patients.

In summary, this study demonstrated that glucocerebrosidase activity has a role in modulating α-synuclein homeostasis and PD-associated behaviors. Collectively, our results provide *in vivo* evidence that augmenting glucocerebrosidase activity in the CNS is a potential disease-modifying strategy for patients with PD, regardless of the mutation status of *GBA1*.

## Materials and Methods

### Animals

The Institutional Animal Care and Use Committees at Genzyme, a Sanofi Company, and the University of California, San Diego, approved all procedures. The *PrP-A53T-SNCA* transgenic mice were engineered to express mutant human A53T α-synuclein (line M83) under the transcriptional control of the murine PrP promoter (31). The *Thy1-SNCA* transgenic mice express WT human α-synuclein (line 61) under the transcriptional control of the murine Thy1 promoter (41).

### Partial inhibition of glucocerebrosidase activity in WT and *PrP-A53T-SNCA* mice using CBE

A partial reduction in CNS glucocerebrosidase activity in WT and *PrP-A53T-SNCA* mice (animals with WT alleles of *Gba1*) was achieved by intraperitoneal administration of a covalent inhibitor of the enzyme, CBE (100 mg/kg, *ip*, 3 times per week for 10 weeks). Daily administration of CBE has been reported to result in a murine model that mimics the enzyme deficiency in Gaucher patients (67). Preliminary studies indicated that an administration interval of 48–72 h was optimal to achieve a residual brain glucocerebrosidase activity close to 50%. Single administration of CBE (100 mg/kg, *ip*) in WT mice resulted in a CNS residual activity of 28 ± 3% after 24 h; 42 ± 2% after 48 h; and 57 ± 3% after 72 h (*n* = 4/group). Consistently, administration of two successive injections of the irreversible inhibitor at corresponding intervals resulted in equivalent reductions (CNS residual activity: 31 ± 3% after 24/24 h; and 42 ± 1% after 48/48 h). Therefore, in order to achieve a partial reduction in CNS glucocerebrosidase activity a three times per week dosage was adopted.

Animals were randomized into two groups and were administered either vehicle or CBE over the trial period. Several assays for motor function and cognition were then performed on the animals starting at 8 weeks post-treatment. All tissues were collected ∼3 h after the last CBE injection; therefore the reported hydrolase activities and lipid quantifications represent the higher levels of pharmacological inhibition achieved.

### Behavioral tests

#### Pole test

The pole test was used to assess basal ganglia-related movement disorders in mice (68,69). In brief, animals were placed facing upwards on top of a vertical wooden pole. The time required for the animals to orient themselves into the downward position and to descend the length of the pole was recorded (68,69).

#### Nest-building test

Orofacial shredding to build nests was used to assess nigrostriatal sensorimotor function in the mice (69). This behavior requires the use of orofacial and forelimb movements, as the animals pull the nesting material apart with their forelimbs and teeth and subsequently break down the material in their mouths and incorporate it into their bedding. In brief, a 3-g nestlet (Pharmaserv, Framingham, MA) was placed into the feeder of the cage of individually housed mice. Pulling the cotton from the feeder requires the mice to rear up and to exercise complex fine motor skills. Nest-building scores were assessed, and the amount of unused nestlet material was measured after 16 h as an indicator of the nigrostriatal sensorimotor function of the mice (34).

#### Rotarod test

The balance and motor coordination of mice were tested by accelerating rotarod as previously described (70). In brief, the mice were placed on the rotarod (Ugo Basile, Italy), which accelerated from 0 to 60 rpm over 300 s. The latency to fall was recorded over three trials.

#### Fear conditioning test

Cued and contextual fear memories were assessed by measuring the freezing responses using the Near-Infrared Fear Conditioning System (Med Associates, VT) as previously described (26). In brief, the mice were trained with a three-trial delay-cued protocol. After a 24-h retention period, freezing time (defined as the lack of movement, except for respiration) was recorded when the mice were exposed to the contextual environment presented during the training period. After 1 h, the mice were placed back into the chamber in a novel context to assess their freezing responses to the acoustic cue.

#### Open-field exploration test

The animals’ locomotor and exploratory activities were determined using the Open Field Activity System (Med Associates, VT). Total distance traveled and time spent in the center quadrant of the cage were measured to assess motor activity and anxiety-related behavior (26).

### Fractionation and quantification of α-synuclein levels

The striata of *PrP-A53T-SNCA* mice were homogenized as previously described to generate three fractions of α-synuclein: cytosolic (Tris-soluble), membrane-associated (Triton-X100-soluble) and insoluble (SDS-soluble). The concentration of human α-synuclein in the different fractions was quantified by sandwich ELISA (Invitrogen, Carlsbad, CA). The protein concentration was determined by the microBCA assay (Pierce, Rockford, IL).

### Generation of recombinant AAV vectors

Recombinant self-complementary AAV2/1-GusB-hGBA1 (AAV1-GBA1) and scAAV2/1-GusB-empty (AAV1-EV) vectors were prepared by triple transfection and purified by ion-exchange chromatography as previously described (26). The titers of the resulting vector preparations were 8e12 gc/ml for AAV1-EV and 7e12 gc/ml for AAV1-GBA1. The viral vectors were injected bilaterally into the striatum of 2-month-old female *Thy1-SNCA* transgenic mice (*n* = 16 per group, 2.5 μl/site, coordinates A/P +0.50, M/L ± 2.00, D/V −2.50).

### Measurement of glucocerebrosidase and hexosaminidase activities and glycosphingolipid levels

Brain glucocerebrosidase and hexosaminidase activities were determined using the artificial substrates 4-methylumbelliferyl (4-MU)-β-d-glucoside or 4-MU-*N*-acetyl-β-d-glucosaminide as previously described (26). Tissue glucosylceramide C18:0 (GlcCer) and glucosylsphingosine (GlcSph) levels were measured by mass spectrometry as previously described (26).

### Immunocytochemical and neuropathological analysis

Immunohistochemical staining for α-synuclein, glucocerebrosidase, DAT and TH was performed in serially sectioned, free-floating vibratome sections. The sections were incubated overnight at 4°C with a polyclonal antibody against total α-synuclein (1:500, affinity-purified rabbit polyclonal antibody; Millipore) (71), glucocerebrosidase (mouse monoclonal antibody; Abnova, Walnut, CA), TH (mouse monoclonal antibody; Millipore, Billerica, MA) or DAT (mouse monoclonal antibody; Millipore, Billerica, MA) followed by a secondary biotinylated antibody (1:100, Vector Laboratories, Inc., Burlingame, CA) and Avidin D-HRP (1:200, ABC Elite, Vector). Detection was performed with 3,3′-diaminobenzidine (72). A subset of sections that were immunostained with the α-synuclein antibody was subjected to proteinase K pre-treatment (8 min, 10 µg/ml). All sections were processed simultaneously using the same conditions. Immunostained slides were analyzed with a digital Olympus bright field digital microscope (BX41). For each animal, a total of three sections (four digital images per section at 400× magnification) were obtained from the striatum and analyzed as previously described with the ImageJ program (NIH) (71).

### Quantitative real-time PCR (TaqMan) analysis

In brief, striatal mRNA was extracted using the RNeasy mini kit (Qiagen, Valencia, CA) and then reverse transcribed and amplified with the TaqMan One-Step RT-PCR master mix kit (Life Technologies, CA) according to the manufacturer’s instructions. The relative mRNA levels of the target genes were determined by quantitative real-time PCR using a microfluidic card TaqMan^®^ Low Density Array (TLDA) on an ABI Prism 7500 (Life Technologies, CA). The expression levels were normalized using DataAssist v2.0 Software (Life Technologies, CA). The endogenous controls used for analysis were β-actin, GAPDH, hypoxanthine-guanine phosphoribosyltransferase-1 (Hprt1) and mouse 18S mRNAs.

### Statistical analysis

Statistical analyses were performed by Student’s *t-*test or analysis of variance (ANOVA) followed by the Newman–Keuls post hoc test. All statistical analyses were performed with GraphPad Prism v4.0 (GraphPad Software, San Diego, CA). Values of *p *< 0.05 were considered significant.
